# Exploring the Reactivity of the CH Radical toward Nitrous Oxide in the Context of the Interstellar Medium

**DOI:** 10.1021/acs.jpca.6c02075

**Published:** 2026-07-01

**Authors:** Daniel I. Lucas, Madeleine E. Robertson, John R. Scott, Lok Yiu Wu, Théo Guillaume, Dwayne E. Heard, Julia H. Lehman

**Affiliations:** † School of Chemistry, 1724 University of Birmingham, Edgbaston, United Kingdom B15 2TT; ‡ School of Chemistry, 4468 University of Leeds, Leeds, United Kingdom LS2 9JT

## Abstract

Experimental measurements of the kinetics of the CH + N_2_O reaction are reported for the first time for the temperature range of 32(3) – 110(4) K using the recently commissioned highly instrumented low temperature reaction chamber (HILTRAC). Furthermore, we report the characterization of a new Laval nozzle to achieve uniform supersonic flow (USF) temperatures of 73(3), 86(4), and 110(4) K with an argon buffer gas. CH radicals generated from photolysis of CHBr_3_ at 248 nm were detected by laser-induced fluorescence using the CH B ^2^Σ^–^ ← X ^2^Π (1,0) Q_2_(1) transition near 364 nm, measuring the pseudo-first-order rate coefficients in the presence of N_2_O. From these experiments, the reaction rate coefficient at 32(3) K was measured to be 1.7(1) × 10^–10^ cm^3^ molecule^–1^ s^–1^ and is at least a factor of 2 greater than the previously measured value at room temperature. The reaction rate coefficient was found to exhibit a positive temperature-dependence below 50 K, while exhibiting a negative temperature-dependence at higher temperatures. We also report the reaction potential energy surface for this reaction, performing *ab initio* calculations at the CCSD­(T)/aug-cc-pV­(Q+d)­Z//M06–2X-D3/aug-cc-pV­(Q+d)­Z level of theory. From this, we identified reaction pathways leading to exothermic product channels for NO + HCN, NO + HNC, N_2_ + HCO, and N_2_ + H + CO, suggesting that NO + HCN are the primary reaction products due to the barrierless nature of this reaction pathway. Finally, a modified Arrhenius fit to all experimental data (32–1300 K) yields *k*(*T*) = (9.3(4) × 10^–11^) × (*T*/300)^−1.03(6)^ exp(−52(5)/*T*) cm^3^ molecule^–1^ s^–1^, which can be incorporated into astrochemical models to better understand the nitrogen-based chemistry of the interstellar medium.

## Graphical abstract

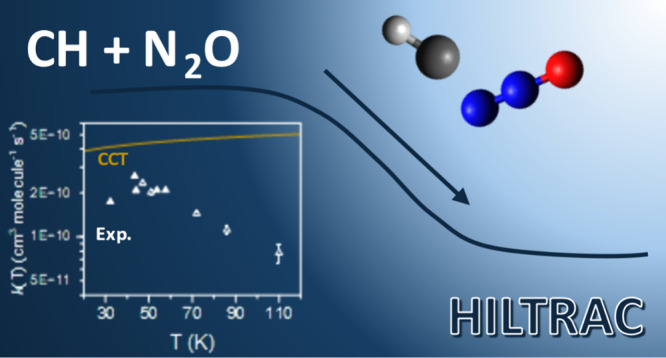

## I Introduction

Molecules containing nitrogen are ubiquitous in the interstellar medium (ISM), with around 140 confirmed detections of nitrogen-bearing molecules in interstellar and circumstellar environments.^1^ For example, the prebiotic molecules formamide (NH_2_CHO), acetamide (NH_2_COCH_3_), *N*-methylformamide (CH_3_NHCHO), and urea (NH_2_CONH_2_) have recently been observed in space.^2–5^ Nitrogen has an atomic abundance relative to atomic hydrogen of around 7 × 10^–5^, making it the fifth most abundant element in the Universe, and so observations of complex molecules containing nitrogen may not be so surprising.^6,7^ However, there is a lack of understanding of the formation and destruction pathways of such molecules. One hypothesis for the formation of larger prebiotic molecules in the ISM is that they can be synthesized from smaller nitrogen oxide molecules such as NO, N_2_O, HNO, and HONO.^8^ Indeed, the hydrogenation of NO is thought to produce hydroxylamine (NH_2_OH) and N_2_O as a byproduct.^9–11^ These nitrogen oxide species, particularly NO, have been detected in the ISM and are thought to play a significant role in the interstellar atomic nitrogen budget.^8,12–19^ Therefore, understanding the reactivity of these molecules, particularly the removal of key molecules such as N_2_O, could play a key role in understanding the interstellar nitrogen budget and the formation of prebiotic molecules.

There are very few experimental or theoretical studies of N_2_O reactions under interstellar conditions. Reactions of N_2_O with the carbon-based radical species CN, C_2_H, CH_2_, and CH_3_ radicals were only performed at room temperature or above and were found to have rate coefficients on the order of 10^–14^ cm^3^ molecule^–1^ s^–1^ at the lowest experimental temperature (298 K).^20–23^ With atomic radicals, the reaction of N_2_O with N atoms is spin-forbidden, and the O + N_2_O reaction potential energy surface (PES) possesses a significant activation energy barrier, making these unlikely candidates as sinks for N_2_O in the ISM.^24,25^ In contrast, one reaction that has been explored under interstellar temperature conditions is that of C (^3^P) + N_2_O.^26^ The authors used a combined theoretical and experimental approach to characterize this reaction in which the rate coefficient was found to more than double from 3.4 × 10^–11^ cm^3^ molecule^–1^ s^–1^ at 296 K to 7.9 × 10^–11^ cm^3^ molecule^–1^ s^–1^ at 50 K. Also, the primary reaction product channel was predicted to be that of CN + NO rather than the thermodynamically favored (most exothermic) CO + N_2_ product channel because there is an energy barrier along the CO + N_2_ reaction pathway that lies 22.54 kJ mol^–1^ above the entrance channel.

Another reaction that could affect the astronomical abundance of N_2_O is the CH + N_2_O reaction. The methylidyne radical, CH, is regarded as an important proxy of the H_2_ column density among astronomers and has an abundance of around 2 × 10^–8^ relative to H_2_.^27,28^ Furthermore, the methylidyne radical is known to play a role in carbon-chain growth, leading to the formation of polycyclic aromatic hydrocarbons (PAHs) and the formation of unsaturated complex organic molecules (COMs), impacting the overall carbon budget of the ISM.^29,30^ It is estimated that around 20% of galactic carbon is contained within PAHs or their derivatives.^31^ What makes the CH radical interesting is that the ‘electron-deficient’ nature of the radical facilitates reactions with other molecules via a barrierless insertion or addition mechanism, meaning reactions of this radical are important in the ISM.^29,32,33^ In fact, the methylidyne radical is so reactive that CH is one of the few radicals that can react with molecular nitrogen.^34–36^ Since both CH and N_2_O are found with appreciable abundances in the ISM, this reaction warrants investigation to examine the impact this may have in such astrophysical environments.

Many possible exothermic product channels have been identified, expanding on those reported by Wagal et al.,^36^ from enthalpies of formation at 0 K for the CH + N_2_O reaction, which are represented by R1–R15.
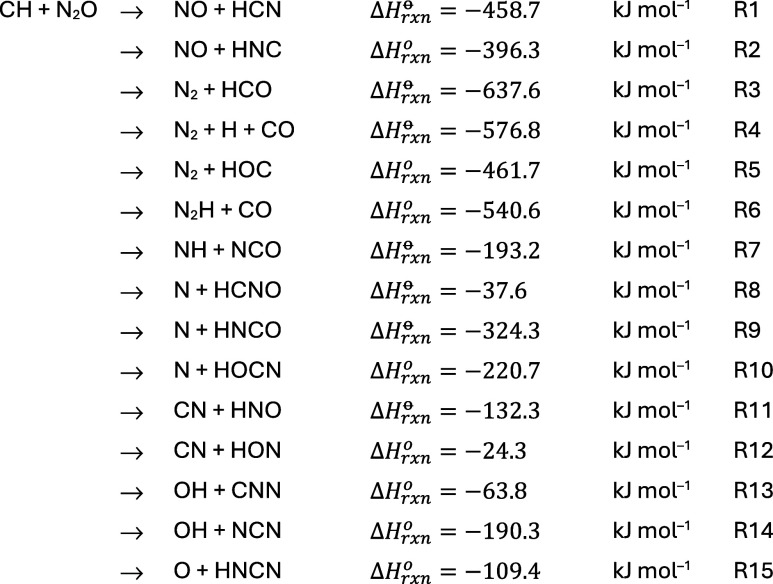


Considering the high reactivity of the CH radical and that most of the product species have been firmly identified in the ISM, the CH + N_2_O reaction could provide an essential gas-phase destruction route of nitrous oxide.^37–47^ No prior theoretical work has been performed to characterize the reaction PES. However, this reaction has been studied experimentally, albeit at temperatures above 200 K and not at temperatures relevant to cold regions of the ISM. Zabarnick et al. have studied this reaction between 297 and 669 K, while Becker et al. reported reaction rate coefficients between 199 and 1300 K.^48,49^ In addition, Wagal et al. reported the reaction rate coefficient at 300 K only, while Anderson et al. reported the reaction rate coefficient from a fast flow study at 290 K only.^36,50^ Good agreement is observed below 500 K; however, there are significant deviations in the trend of the reaction rate coefficients at elevated temperatures. No pressure dependence was observed in these experimental studies. Further to this work, Hovda and Hershberger used tunable diode lasers to detect reaction products at room temperature. They reported that R1 is the primary product pathway, with a branching percentage of 72%, while R4 is the minor product pathway at 28%.^51^ However, they were unable to detect CN and HCO. The authors also found evidence of HNCO and HCNO in static gas IR spectra but were unable to quantify the branching fraction for these product channels.

With this in mind, the goal of this work is to experimentally examine the gas-phase reaction of CH + N_2_O, supported by *ab initio* electronic structure calculations exploring the reaction potential energy surface. The HILTRAC apparatus, based on a pulsed CRESU expansion, was used to measure reaction rate coefficients between 32(3) and 110(4) K.^52^ The pulsed laser photolysis – laser-induced fluorescence (PLP-LIF) detection technique was employed to generate CH radicals from bromoform and to monitor their decay in the presence of N_2_O. Finally, the trend in the value of *k*(*T*) as a function of reaction temperature was examined, making links to the calculated reaction PES and to other CH radical reactions.

## II Methodology

### A Experimental Measurements

Kinetic measurements were made using the PLP-LIF technique in the HILTRAC apparatus described in detail elsewhere.^52^ Briefly, a gas mixture containing CHBr_3_ (Sigma-Aldrich 99%), N_2_O (BOC, 99.99%), and an argon buffer gas (BOC, 99.99%) was pulsed into a reservoir (∼35.1 cm^3^), which subsequently undergoes supersonic expansion through a Laval nozzle. This produced a uniform supersonic flow (USF) with a total gas density of 4.3(6) × 10^16^ molecules cm^–3^ at 32(3) K, primarily composed of an argon buffer gas and entraining typical CHBr_3_ and N_2_O densities of approximately 5.9(9) × 10^12^ molecules cm^–3^ and 1.0(2) to 15.1(24) × 10^13^ molecules cm^–3^, respectively. To generate CH radicals along the isentropic core of the USF, a KrF excimer laser (Coherent COMPex 102) was directed coaxially with the USF to photolyze CHBr_3_ at 248 nm (4 mm diameter spot size, 70 mJ per pulse after the Laval nozzle).^53,54^ It is assumed that CH radicals are initially generated uniformly by the photolysis laser along the region of the USF used for kinetics studies. To detect the CH radicals, a UV probe laser was oriented perpendicular to the flow direction and spatially overlapped with both the uniform flow and excimer laser, and positioned 10 to 20 cm downstream from the nozzle exit. The exact distance depends on the length of uniformity of the USF, which differs for each Laval nozzle used. The UV probe laser was the frequency-doubled output from a dye laser (Sirah Cobra Stretch, LDS722 dye) pumped by the 532 nm output of an Nd:YAG laser (Continuum 8000, 10 Hz). A laser spot size of 2 mm and pulse energy of 2 mJ was typically used. The CH radicals were excited by the probe laser using the CH B ^2^Σ^–^ ← X ^2^Π (1,0) Q_2_(1) transition at 363.432 nm.^54,55^ The resulting B ^2^Σ^–^ → X ^2^Π (1, 1) fluorescence was detected using a PMT mounted orthogonally to the plane of the photolysis laser and probe laser, with a bandpass filter (Semrock, FP01–405/10–25) centered at 405(10) nm.

The relative delay time between the photolysis and probe laser pulses for each gas pulse was randomly varied to reduce effects of laser power fluctuations and slow drifts impacting the signal. The delay times varied from −20 to 200 or 300 μs, depending on the maximum available kinetic time for each Laval nozzle, such that both the photolysis and probe laser pulses overlap spatially and temporally at 0 μs. Here, the CH signal results from CH generated at the fluorescence detection axis. In the experiments, the nozzle position was such that the fluorescence signal observed at the longest time delay was from nascent CH generated at the exit of the Laval nozzle and thus corresponds to the time required for the CH radical to reach the fluorescence detection axis. Each kinetic decay comprises up to 160 data points, with a 2 μs spacing between the laser delay times. At each laser delay time, the fluorescence decay traces of the CH radical were averaged five times to increase the signal-to-noise ratio on the resulting kinetic decay trace while balancing the time required for data acquisition. For each laser delay time, the averaged fluorescence trace was digitized and transferred to a computer for processing using an in-house Python script.

A single exponential decay function was fit to the averaged fluorescence decay signals, and integrated to obtain a value proportional to the relative amount of CH in the flow for a given laser delay time, [CH]_*relative*_. Reactions were performed under pseudo-first-order conditions by holding the N_2_O density in the USF in excess of the CH radical. The pseudo-first-order rate coefficient was obtained from a single-exponential fit to a plot of [CH]_*relative*_ versus time for the specific excess concentration of N_2_O in the USF, as seen in [Disp-formula eq1], which is dependent on the pre-exponential factor, *A*, and the pseudo-first-order rate coefficient, *k′*. Before fitting, the kinetic decay profiles were baseline-corrected by averaging the first 14 μs at negative laser delay times and subtracting this value from the rest of the kinetic decay trace. To negate any effects of PMT gating or rotational and/or vibrational relaxation of excited CH radical directly after photolysis of bromoform, which may result in nonexponential decay, kinetic decay profiles were fit from several laser delay times between 20 and 36 μs relative to *t* = 0 (photolysis and probe lasers temporally overlapped). The resultant CH loss rates due to reaction with N_2_O were found to be independent of the fit starting point, suggesting the single exponential decay fitting parameter is free from CH rovibrational relaxation or PMT gate interference after 20 μs. Thus, a single exponential decay function given by [Disp-formula eq1] was fit to the integrated fluorescence signal for time scales longer than 30 μs.

E1 $${\left[\mathrm{CH}\right]}_{relative}=A\;\exp\left(-k^{\prime }t\right)$$

The above process was then repeated for multiple excess N_2_O concentrations, adjusting the Ar flow rate to compensate for changes in the overall density of the USF in order to maintain USF conditions. The temperature-dependent reaction rate coefficients for CH + N_2_O were then calculated from the slope of a linear fit of the pseudo-first-order reaction rate coefficient as a function of N_2_O concentration. Each measured second-order rate coefficient is for the specific temperature of the USF generated by the Laval nozzle. Here, the reaction rate coefficients were measured across multiple temperatures between 32(3) and 110(4) K by changing the Laval nozzle and/or conditions of the flow to yield a different temperature and then repeating the above procedure to derive the reaction rate coefficient at the new temperature. The temperatures for the Laval nozzles used are derived from impact pressure measurements and confirmed by rotational temperature using frequency comb spectroscopy or LIF excitation spectral fitting, as shown in the Supporting Information for selected conditions and discussed previously.^52,56–59^ The propagation of error was discussed extensively in a previous publication, and uncertainties in parentheses denote one standard deviation in the final reported digit.^52^

### B Electronic Structure Calculations

The calculations presented here were performed using the Gaussian16 software package with the M06-2X-D3 functional and the correlation-consistent aug-cc-pV­(Q+d)­Z Dunning basis set.^60–62^ Stationary point structures (reactants, products, intermediates, and transition states) along the PES were first optimized using the very tight (opt = verytight) convergence criterion and an ultrafine DFT grid (int = ultrafine). Following this, harmonic vibrational frequencies of the successfully optimized structures were calculated. Transition state species were identified as those that possess a single imaginary vibrational frequency, while minima were identified as those possessing all real vibrational frequencies. All calculated vibrational frequencies were scaled by the appropriate scaling factor for the chosen level of theory of 0.972.^60^ Transition states were then subsequently confirmed through IRC calculations, and stationary points along the reaction PES were identified through exploring relaxed scans of the entrance channel as CH approaches N_2_O. Once all structures were optimized, single point energy calculations were performed at the CCSD­(T)/aug-cc-pV­(Q+d)­Z level of theory to refine the calculated electronic energy. Significant multireference character was not anticipated for this doublet PES, and no evidence of problematic behavior was observed during the calculations. Calculated properties (Cartesian coordinates, electronic energies, rotational constants, and vibrational frequencies) for all optimized structures are provided in the Supporting Information.

## III Results and Discussion

Reaction rate coefficients for CH + N_2_O were measured over the temperature range 32(3) – 110(4) K using the HILTRAC apparatus in combination with the PLP-LIF technique. Representative temporal profiles of the integrated fluorescence signal, [CH]_*relative*_, recorded at three different N_2_O concentrations are presented in Figure 1. In each case, the integrated CH fluorescence intensity decays exponentially following photolysis, with the observed pseudo-first-order decay rate increasing with N_2_O concentration. For example, the fitted pseudo-first-order rate coefficient rises from 5,500(300) s^–1^ at an N_2_O density of 1.0(2) × 10^13^ molecules cm^–3^ to 15,400(300) s^–1^ at [N_2_O] = 6.8(13) × 10^13^ molecules cm^–3^. Additional checks of the single exponential decay fitting procedure shown in Figure 1 were carried out to ensure their robustness, and these details are given in the Supporting Information.

**Figure 1:**
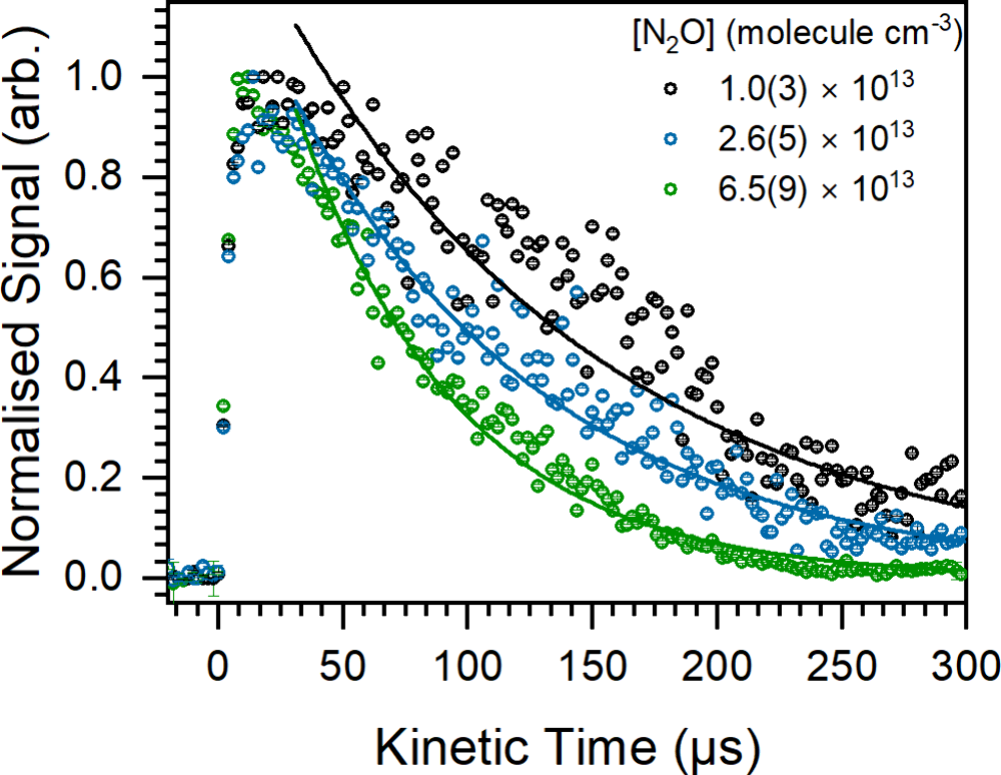
Representative normalized CH integrated fluorescence decay traces obtained at 32(3) K in an Ar flow with a total density of 4.3(6) × 10^16^ molecules cm^–3^. Single exponential decay fits are shown for [N_2_O] = 1.0(3) × 10^13^ (black), 2.6(5) × 10^13^ (blue), and 6.5(9) × 10^13^ (green) molecules cm^–3^.

For each flow temperature, bimolecular reaction rate coefficients were extracted from the dependence of the pseudo-first-order decay coefficient on the N_2_O concentration according to 2:

E2 $$k^{\prime }=k_{\mathrm{CH}+N_{2}O}\left[N_{2}O\right]+k_{0}$$

where *k*_CH+N_2_O_ corresponds to the bimolecular reaction rate coefficient for reaction with N_2_O and *k*_0_ accounts for background CH loss processes, such as diffusion out of the detection zone, or reactions with precursor species and possible secondary photolysis products. A representative bimolecular plot obtained using Nozzle 9, which produces a 32(3) K USF when using Ar as the buffer gas, is shown in Figure 2. At lower N_2_O concentrations, the pseudo-first-order rate coefficient varies linearly with N_2_O density, as expected under pseudo-first-order conditions. However, once the N_2_O density exceeds approximately 1.1 × 10^14^ molecules cm^–3^, a clear deviation from linearity becomes apparent and the observed decay rate begins to level off. This behavior is attributed to clustering of N_2_O under the cold flow conditions, most likely through dimer or oligomer formation, thereby reducing the effective monomer concentration available for reaction. Consequently, only the linear low-density region was included in the least-squares regression analysis used to determine the bimolecular rate coefficient.

**Figure 2:**
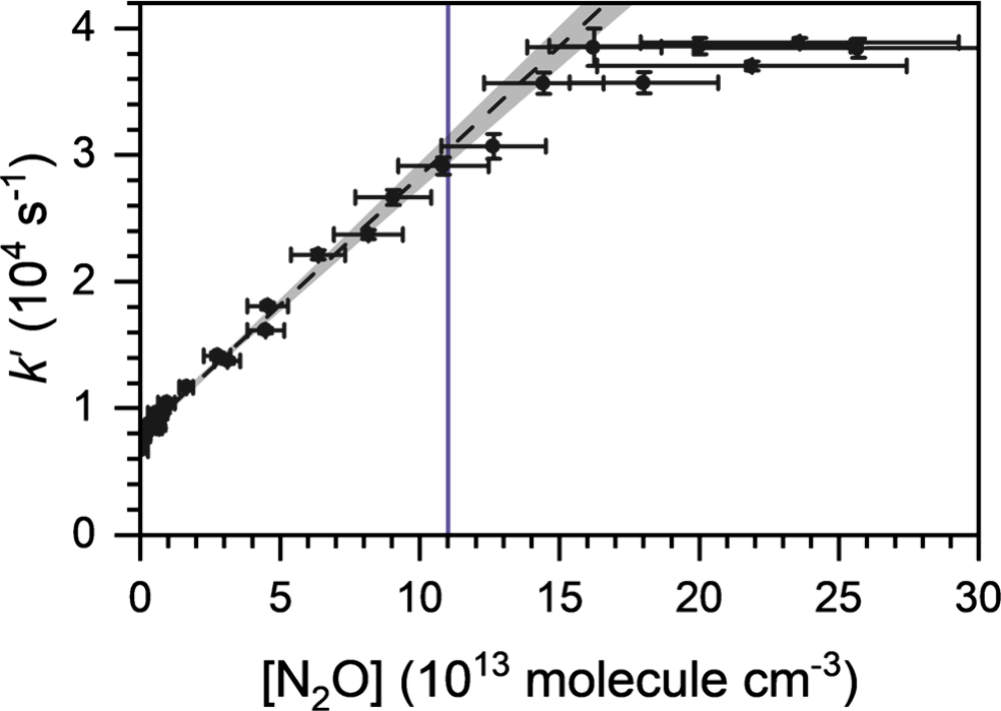
Pseudo-first-order CH loss rates measured as a function of N_2_O density at 32(3) K in an Ar flow with a total density of 4.3(6) × 10^16^ molecules cm^–3^ and [CHBr_3_] = 4.5(5) × 10^12^ molecules cm^–3^. The solid line represents the linear least-squares regression fit used to derive *k*(32­(3) K) = 1.9(1) × 10^–10^ cm^3^ molecule^–1^ s^–1^, while shaded regions indicate the 95% confidence bands. Due to the observation of curvature at high N_2_O concentrations, data for [N_2_O] > 1.1 × 10^14^ molecules cm^–3^ (purple line) were excluded from the fit.

From repeated measurements at 32(3) K, an average rate coefficient of 1.9(1) × 10^–10^ cm^3^ molecule^–1^ s^–1^ was obtained, together with an intercept value of 7,900(200) s^–1^ for an average total density of 4.3(6) × 10^16^ molecules cm^–3^. Nonzero intercepts (*k*_0_) are commonly observed in CRESU and flow cell kinetics studies and are generally associated with background radical loss processes.^54,63–66^

To assess reproducibility, measurements at 32(3) K were repeated nine times, while a minimum of three independent data sets were acquired at all other temperatures. The derived rate coefficients were also found to be insensitive to both the precursor concentration and the specific rovibronic transition monitored. In particular, varying the CHBr_3_ concentration between 3 × 10^12^ molecules cm^–3^ to 6 × 10^12^ molecules cm^–3^ produced no systematic change in the intercept term *k*_0_. Although reactions involving precursor fragments or photolysis byproducts may contribute to background CH removal, these observations suggest that diffusion from the detection region is likely the dominant source of the intercept. Seven different Laval nozzles, four machined and three 3D printed, were used to measure reaction rate coefficients between 32(3) and 110(4) K. Typically, nitrogen can be used as an alternative buffer gas in order to reach higher temperatures in CRESU experiments, but as discussed previously,^52^ CH reacts with molecular nitrogen, and so the kinetic results can be exceedingly difficult to interpret. To extend experimental measurements of *k*(*T*) up to 110(4) K using only an Ar buffer gas, new Laval nozzles were designed by collaborators at the University of Leeds.^59^ Representative bimolecular plots for all machined nozzles and all measured reaction rate coefficients can be found in the Supporting Information.

The experimental temperature dependence of the reaction rate coefficient is shown in Figure 3. Within the temperature range 32(3) < *T* < 110(4) K, *k*(*T*) exhibits a slight positive temperature dependence followed by a negative temperature dependence, peaking around 50 K. The negative temperature dependence is consistent with significant contribution from a barrierless capture process, which is not unsurprising for the highly reactive CH radical. No detailed examination of the pressure dependence was performed in this study. To further examine the trend in *k*(*T*), the experimental reaction rate coefficients were compared to those calculated by classical capture theory (CCT),^67–69^ and the previously published experimental works at higher temperature in Figure 3. CCT provides an upper estimate to the overall reaction rate coefficient. The CCT calculations consider all intermolecular forces, exclude short-range interactions, and assume all collisions yield products. These calculations were performed using values from the literature by the methods described in Section B of the Supporting Information.^70–75^ It predicts a 2-fold decrease in the upper limit of *k*(*T*) from 7.2 × 10^–10^ cm^3^ molecule^–1^ s^–1^ at 1000 K to 3.6 × 10^–10^ cm^3^ molecule^–1^ s^–1^ at 10 K, without indicating a negative temperature dependence. Given N_2_O has a dipole moment ≪ 1 D, CCT suggests that the long-range attraction of CH and N_2_O is dominated by London dispersion forces since *C*_*6*_^*Disp*^ > *C*_*6*_^*D‑D*^ down to 10 K. Specific details of the CCT calculations are provided in the Supporting Information.

**Figure 3:**
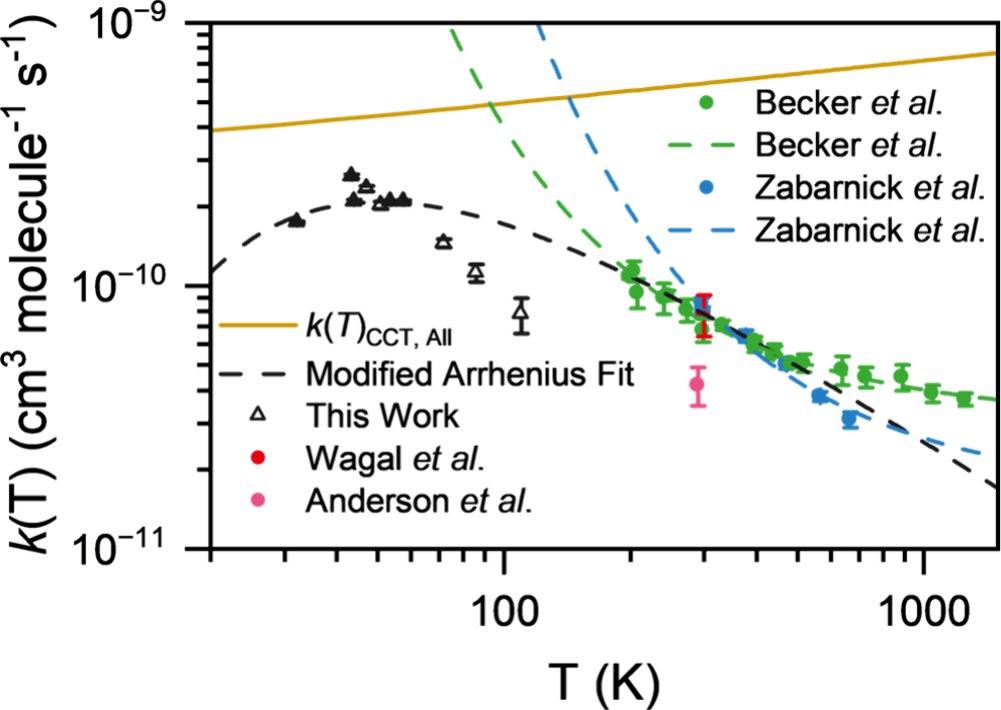
Averaged values of *k*(*T*) for CH + N_2_O as a function of temperature measured in this work (black hollow triangles), compared to experimental values of Zabarnick et al.^48^ (blue), Becker et al.^49^ (green), Wagal et al. (red solid circle), and Anderson et al. (pink circle). The black dashed line represents a modified Arrhenius fit. The errors represent one standard deviation of the average value. The overall rate coefficient calculated by collision capture theory is shown as a solid gold line.

Figure 3 also illustrates previously published Arrhenius fits to the experimental rate coefficients from Zabarnick et al. and Becker et al. as blue and green dashed lines. When extrapolated beyond the previous experimental measurements, the fit extends to unrealistically high values of *k*(*T*) below 100 K, exceeding the CCT limit.^48,49^ Such an extrapolation that yields unrealistic values underscores the need for caution when extending the fit beyond the previously measured temperature ranges and highlights the need to include low-temperature experimental measurements or theoretical calculations. A global fit of a modified Arrhenius function ([Disp-formula eq3]) to the lower temperature experimental data plus the previous higher temperature data results in a more appropriate representation of the combined experimental data. A piecewise modified Arrhenius expression would likely provide a more physically representative description of the experimental trends across the full temperature range, but the single functional form captures the general trend of the *k*(*T*) data set while also being easily reported in astrochemical databases and implemented in astrochemical models.

k(T)=α×(T300)β×e−(γT) E3

The functional form used here is the same as that used in the KIDA database, where the parameters translate as α being the pre-exponential factor (cm^3^ molecule^–1^ s^–1^), β is a constant, and γ represents the activation energy divided by the ideal gas constant.^76^ The final fit yields *k*(*T*) = (9.3(4) × 10^–11^) × (*T*/300)^−1.03(6)^ exp(−52(5)/*T*) cm^3^ molecule^–1^ s^–1^. In the work of Zabarnick et al. (*T* > 297 K) and Becker et al. (*T* > 199 K), the authors reported a slight negative temperature dependence.^48,49^ While that trend generally continues to lower temperatures, it transitions to a positive temperature dependence below approximately 50 K, which is captured in the modified Arrhenius fit.

Interestingly, the observed trend of transitioning between a negative and positive temperature dependence has been previously observed for CH radical reactions with a number of different reaction partners, such as organic molecules.^29,54,77–80^ One previous suggestion is that the reactivity of CH toward hydrocarbons, for example, is governed by long-range capture and proceeds over a barrierless potential energy surface. In each case, the rate coefficients approach a collision limit which is determined by the long-range capture forces between the reagents. This is because the relative velocity of molecules decreases with decreasing temperature, even as the collision cross-section increases, causing the decline in the value of *k*(*T*).^29^ However, the overall trends in temperature dependence are likely governed by a combination of long-range capture effects and access to multiple submerged and nonsubmerged regions of the PES, whose relative importance may evolve with collision energy.

A deviation of the experimental data from the modified Arrhenius fit is observed for the three data points at 73 K and above. Experimental values were verified through three repeat experiments at each temperature, the results of which can be found in the Supporting Information. The temperature of the flow was also verified using the LIF excitation spectrum in addition to impact pressure measurements for the USF reported as 110(4) K. Fitting of the LIF excitation spectrum of the CH radical detected in this USF (see Section A.ii and Figure S3 in the Supporting Information) found the rotational temperature to be 112(10) K, which is consistent with the translational temperature derived from impact pressure measurement. This suggests that the observed deviation is not likely to be a result of an erroneous determination of temperature. Alongside the experimental reproducibility of the measurements, previous validation of the HILTRAC flow characterization through agreement between experiment and CFD simulations^59^ suggests that the observed deviation is unlikely to arise from an obvious systematic error in the total flow conditions. A systematic scaling factor error in the N_2_O flow rates (concentration determination) would be expected to affect the magnitude of all measured rate coefficients rather than selectively suppressing only the higher-temperature measurements.

Despite the relatively minor deviation from the modified Arrhenius fit, the overall large rate coefficient of CH + N_2_O and approach to the collision capture limit are consistent with a barrierless process, as expected for the majority of CH radical reactions. To confirm this hypothesis, a brief exploration of the reaction potential energy surface was undertaken. Shown in Figure 4 are four reaction pathways found by *ab initio* methods for the approach of CH toward N_2_O. The energies are the zero-point vibrational energy (ZPVE) corrected electronic energy values in kJ mol^–1^ relative to the CH + N_2_O entrance channel. A full exploration of the PES was not undertaken here and instead focused on pathways to the most exothermic products (R1-R4) only.

**Figure 4:**
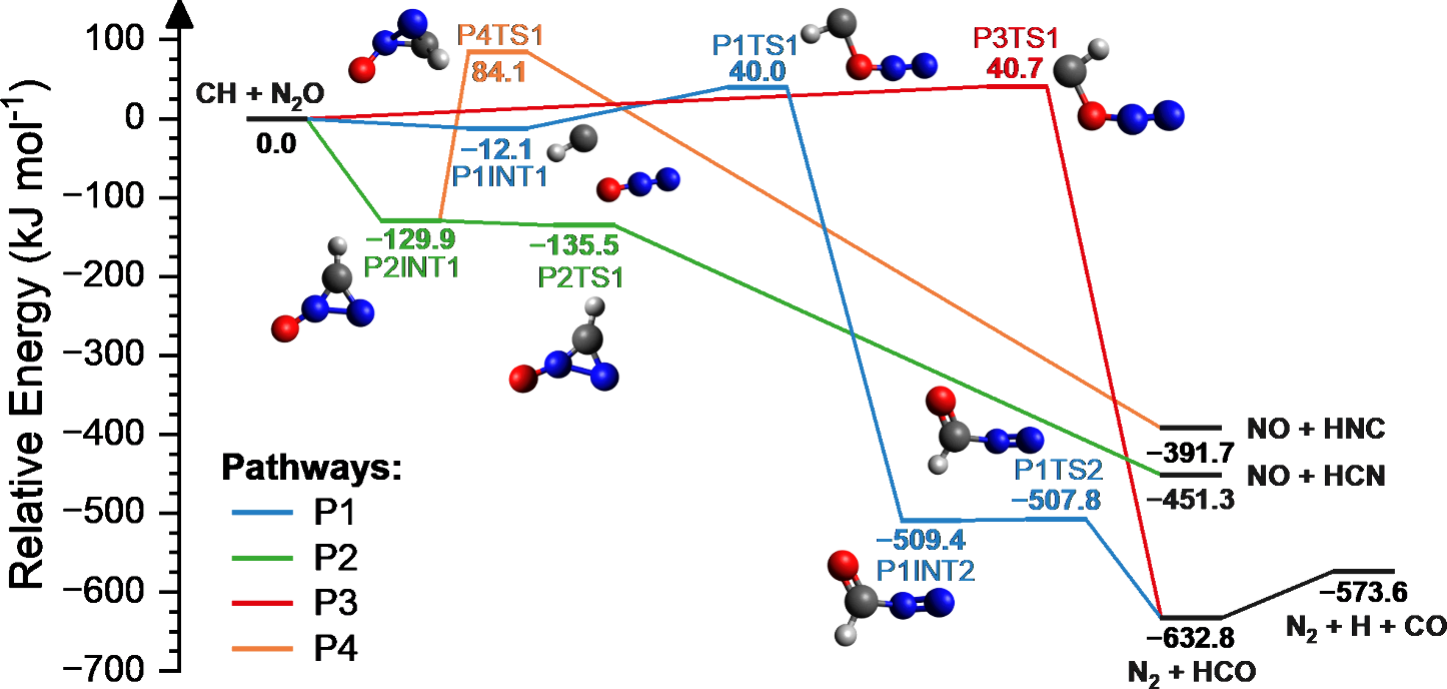
Reaction pathways for the approach of CH toward N_2_O calculated at the CCSD­(T)/aug-cc-pV­(Q+d)­Z//M06–2X-D3/aug-cc-pV­(Q+d)­Z level of theory. Electronic energies (corrected with scaled ZPVE) are quoted in kJ mol^–1^ relative to reactant species. Formation of N_2_ + HCO is shown via P1 (blue) and P3 (red). Formation of NO + HCN is shown via P2 (green), and formation of NO + HNC is shown via P4 (orange). Subsequent dissociation of HCO to H + CO is also indicated in black.

For R1 and R2, one reaction route to NO + HCN (P2, green) and one to NO + HNC (P4, orange) through an H atom migration reaction of P2INT1 was found. The route to NO + HCN is barrierless with respect to the reactants. It proceeds via an addition of CH across the N–N bond, distorting N_2_O away from its linear structure and forming the cyclic intermediate P2INT1, then falling apart into NO + HCN. The rearrangement to HNC via P4TS1 results in a pronounced barrier (84 kJ mol^–1^) above the reactant energies, rendering this pathway inconsequential to the low temperature conditions used in the experiment here.

For R3, forming N_2_ + HCO, two reaction pathways (P1, blue; P3, red) were found: one direct pathway via P3TS1 and one indirect route through P1INT1 and P1INT2. Both proceed via the approach of CH to the oxygen end of N_2_O, in either a trans (P1) or cis (P3) configuration. CH can either insert into the N–O bond before undergoing decomposition to HCO + N_2_ (P1), or undergo O atom abstraction to yield the same result (P3). The insertion route initially proceeds through a van der Waals complex (P1INT1, −12.1 kJ mol^–1^) before inserting into the N–O bond (P1INT2, −509.4 kJ mol^–1^) via a 40.0 kJ mol^–1^ barrier to insertion. There is a similarly large 40.7 kJ mol^–1^ barrier to O atom abstraction via T3TS1. Once N_2_ + HCO is formed, a further breakup of HCO to form H + CO yields the products for R4, N_2_ + H + CO. Given the large barriers along P1 and P3, neither pathway is likely relevant to the low temperatures discussed here, and so R3 and R4 are unlikely to be observed.

From this work, NO + HCN (R1) is expected to be the primary reaction pathway, despite the N_2_ + HCO (R3) and N_2_ + H + CO (R4) product channels holding greater exothermicities. The barrierless process via P2 is consistent with the large reaction rate coefficients experimentally observed across the wide range of temperatures in this and previous work. Interestingly, the previous work on product identification for the title reaction at room temperature indicated a 72%: 28% split between R1 and R4.^51^ Given the calculations performed here, a barrierless (or low barrier) pathway to R4 was not found, and so we would not anticipate a significant fraction of N_2_ + H + CO products. However, we only considered the breakup of HCO as the route to R4 via R3, rather than more exotic intermediates such as HN_2_ + CO, breaking apart to N_2_ + H + CO, as was previously suggested.^51,81^ A more exploratory approach to searching for reaction pathways might help identify additional pathways, such as through recently developed software like KinBot or the SCINE software suite.^82,83^

## IV Conclusion

In this paper, the first experimental measurements of reaction rate coefficients between 32(3) and 110(4) K for the CH (X ^2^Π) + N_2_O gas phase reaction have been presented. The reaction rate coefficient was found to exhibit a positive temperature dependence at the lowest temperatures, followed by a negative temperature dependence with increasing temperatures. This trend has been observed several times before in CH radical reactions. From laboratory experiments, *k*(*T*) at 32(3) K was measured to be 1.7(1) × 10^–10^ cm^3^ molecule^–1^ s^–1^ and is at least a factor of 2 greater than the measured value at room temperature. A modified Arrhenius fit to the experimental data yields a functional form to be used in astrochemical databases: *k*(*T*) = (9.3(4) × 10^–11^) × (*T*/300)^−1.03(6)^ exp(−52(5)/*T*) cm^3^ molecule^–1^ s^–1^. With *ab initio* calculations, reaction pathways to four exothermic product channels (Figure 3) were confirmed. These pathways followed a two-step addition–elimination or insertion-elimination reaction mechanism (P2), or a three-step addition-insertion-elimination reaction mechanism (P1) as CH approached the N or O atom of N_2_O, respectively. A single-step O atom abstraction route (P3) was also found, as well as a pathway to NO + HNC through an H atom migration reaction. While the insight into possible branching fractions is not included in the work presented here, the measured reaction rate coefficients and the modified Arrhenius fit in this work should be included in astrochemical models to better understand the nitrogen-based chemistry of the ISM and to determine the relative importance of this gas-phase reaction to carbon- and nitrogen-based chemistry in the ISM.

## Supplementary Material



## Data Availability

Most data are already available in the Supporting Information. For additional data requests, please contact the corresponding author (j.lehman@bham.ac.uk).

## Notes

Calculated geometries, energies, vibrational frequencies, and rotational constants for stationary points and transition states for the CH + N_2_O reaction PES. Additional experimental details (e.g., flow conditions and kinetic measurements) and capture theory calculations (PDF)

## §

LOMA, Université de Bordeaux, CNRS, UMR 5798, 351 cours de la Libération, Talence Cedex, 33405, FR
