# Supplementary material for: Exploring the Reactivity of the CH Radical toward Nitrous Oxide in the Context of the Interstellar Medium
Source: J Phys Chem A. 2026 Jul 1;130(28):5386–94. doi: 10.1021/acs.jpca.6c02075 (PMC13383739; doi:10.1021/acs.jpca.6c02075)
Supplement: Supplementary file 1 [file jp6c02075_si_001.pdf]

Supplementary Information

Exploring the Reactivity of the CH Radical towards Nitrous Oxide in the Context of the Interstellar Medium

Daniel I. Lucas,[a] Madeleine E. Robertson,[a] John R. Scott,[a] Lok Yiu Wu,[a] Théo Guillaume,[a,†] Dwayne E. Heard,[b] and Julia H. Lehman[a,*]

[a]School of Chemistry, University of Birmingham, Edgbaston, United Kingdom, B15 2TT

[b]School of Chemistry, University of Leeds, Leeds, United Kingdom, LS2 9JT

[†]Current address: LOMA, Université de Bordeaux, CNRS, UMR 5798, Talence Cedex, 33405, 351 cours de la Libération, FR

*Corresponding Author: Julia H. Lehman, j.lehman@bham.ac.uk

Table of Contents

Table of Contents **2**
A. Further Information on HILTRAC Experiments **3**
i. Impact Pressure Measurements 3
ii. Rotational Temperature Confirmation 4
iii. Kinetic Measurements 5
B. Capture Theory Calculations **7**
C. Electronic Structure Calculations **8**
References **11**

A. Further Information on HILTRAC Experiments

i. Impact Pressure Measurements

The uniform supersonic flows (USFs) produced by each Laval nozzle have been characterised using a Pitot tube to measure the impact pressure as a function of displacement. The exact details of the procedure are discussed in detail elsewhere, and the interested reader is directed to previous works.[1–4] Three of the four machined Laval nozzles used to explore the kinetics of the CH + N_2O reaction have been characterised previously.[4] However, the fourth Laval nozzle was newly designed and machined for this work to study the kinetics of CH + N_2O at the higher temperatures of 73(3) K, 86(4) K, and 110(4) K within an Ar flow. The results of impact pressure measurements, showing flow temperature as a function of distance between the nozzle exit and the Pitot tube, are shown in Figure S1. The experimental conditions can be found in Table S1.

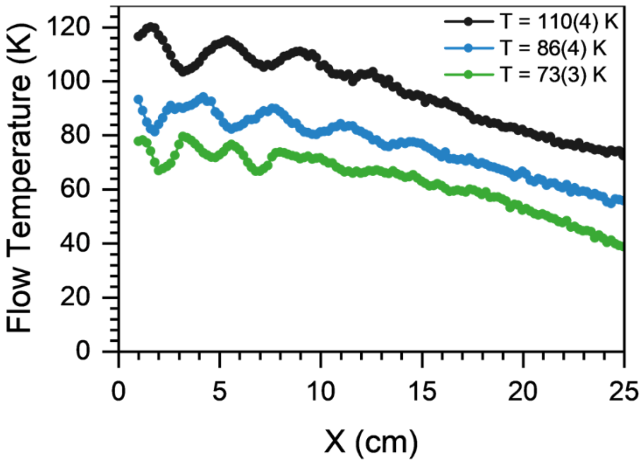

Figure S1: Achievable USF temperature profiles using Nozzle 14 with an Ar buffer gas. The experimental conditions can be found in Table S1. Fluorescence data were collected at 10 cm for each of these USF profiles.

Table S1: Parameters of the USF for Nozzle 14 used in this work with an Ar buffer gas, characterised by impact pressure measurements. The experimental values are the reservoir pressure (P_{res}) and chamber pressure (P_{ch}). The calculated USF properties include the maximum flow length (L), corresponding maximum kinetic time, Mach number, flow pressure (P), flow temperature (T), and flow number density (D).

Experimental Parameters		Calculated USF Properties					
P_{res} (mbar)	P_{ch} (mbar)	L (cm)	Kinetic Time (μs)	Mach	P (mbar)	T (K)	D (10^{16} molecule cm^{-3})
38.9	1.11	10.6	200	3.1(1)	1.1(1)	73(3)	11.3(8)
13.6	0.51	11.6	226	2.7(1)	0.6(1)	86(4)	5.1(4)
15.1	0.55	10.2	214	2.2(1)	1.3(1)	110(4)	8.5(6)

ii. Rotational Temperature Confirmation

For temperature confirmation via rotational fitting of the LIF spectrum, CH radicals were generated and detected using the procedures described in the experimental section of the main text. However, in these experiments, the probe laser wavelength was scanned from 362.5 to 364.5 nm in 0.005 nm increments, keeping the PLP-LIF laser delay time constant at 20 μs which is long enough for rotational relaxation to occur. Each datapoint collected is an average of 10 acquired fluorescence traces. The nozzle was positioned 9 cm from the LIF detection axis, and spectra were collected for probe laser powers of 0.08, 0.2, and 2 mJ pulse^{-1} at a 10 Hz repetition rate. The result for a probe laser power of 0.08 mJ pulse^{-1} is shown in Figure S2, along with a PGOPHER fitting of the spectra. The PGOPHER fitting results in a temperature of 112(10) K, which supports the reported translational temperature of the 110(4) K USF and provides confidence in the reported temperatures of other USFs reported for the same Laval nozzle.

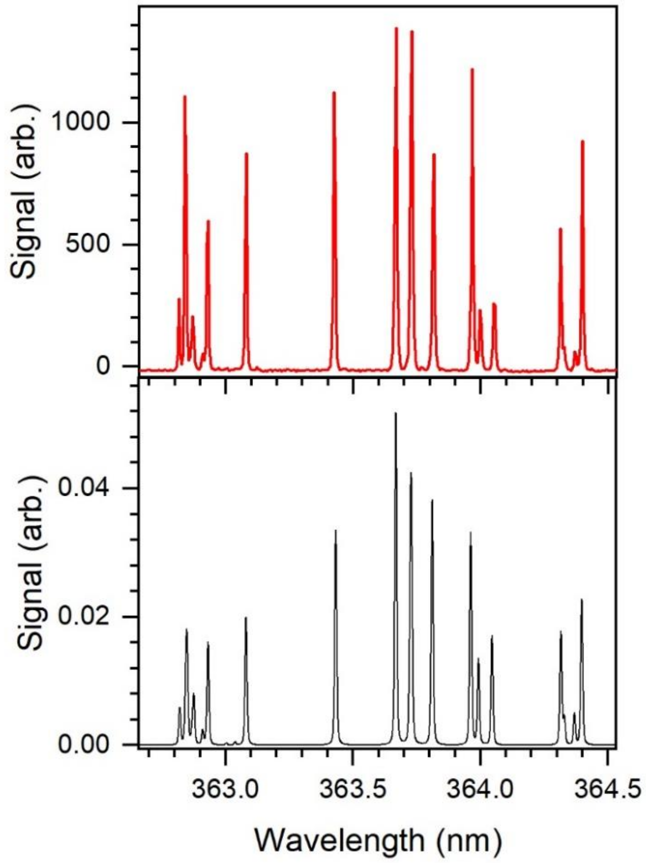

Figure S2: LIF excitation spectra of CH at 110(4) K for a probe laser power of 0.08 mJ pulse^{-1} (top, red) and a PGOPHER fit of the spectrum (bottom, black). Data were collected over the wavelength range of 362.5 to 364.5 nm in 0.005 nm increments at a laser delay time of 20 μs using Nozzle 14 and Ar as the buffer gas.

iii. Kinetic Measurements

As stated in Equation 1 of the main text, the data are fit to the function $[\mathrm{CH}]_{relative} = A\,\exp(-k't)$. However, to ensure the robustness of the single exponential decay fitting procedure, the data and fits in Figure 1 of the main text were transformed via natural logarithm, resulting in the data shown in Figure S3. Here, the propagated uncertainties are given by $\sigma_{+} = \ln(S + \sigma_S) - \ln(S)$ and $\sigma_{-} = \ln(S) - \ln(S - \sigma_S)$, where $[\mathrm{CH}]_{relative}$ in Equation 1 is rewritten as S for brevity, and σ_S is the error in $[\mathrm{CH}]_{relative}$ arising from propagating error in repeated measurements (see Ref. 4 for details).

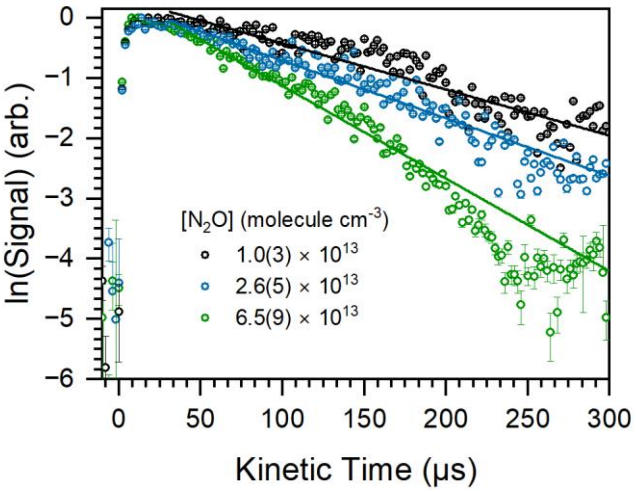

Figure S3: Representative normalised natural logarithm of the transient CH integrated fluorescence signal as a function of laser delay time taken at 32(3) K and a total density in Ar of 4.3(6) × 10^{16} molecule cm^{-3}, together with the natural logarithm of the single exponential decay fits for $[N_2O]$ = 1.0(3) × 10^{13} (black), 2.6(5) × 10^{13} (blue), 6.5(9) × 10^{13} (green) molecule cm^{-3}.

The temperature dependence of the reaction rate coefficient was examined by changing the Laval nozzle, thereby changing the USF temperature. In HILTRAC, changing the USF temperature is possible by switching the buffer gas from Ar to N_2, for example, or by changing the Laval nozzle for the same buffer gas. Since CH is reactive with N_2, only Ar has been used as a buffer gas to measure CH + N_2O reaction rate coefficients.[4–6] Here, seven different Laval nozzles, four machined and three 3D printed, were used to measure reaction rate coefficients between 32(3) and 110(4) K. Representative bimolecular plots for all machined nozzles (excluding that of 32(3) K, which is in the main text) are shown in Figure S4. Each measured reaction rate coefficient is reported in Table S2.

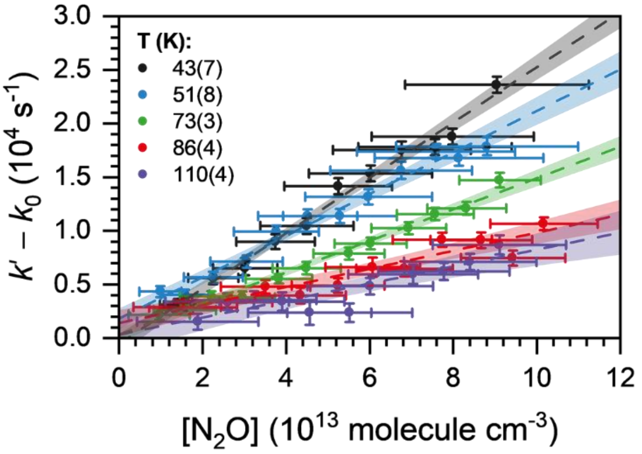

Figure S4: Intercept subtracted pseudo-first-order rate coefficients as a function of N_2O density for USF temperatures of 43(7) (black), 51(8) (blue), 73(3) (green), 86(4) (red), and 110(4) (purple) K using Laval nozzles 12 – 14, respectively. The experimental conditions can be found in Table S2.

Table S2: Measured reaction rate coefficients and experimental conditions for the CH + N_2O reaction.

Gas	T (K)	N_{total} (10^{16} molecule cm^{-3})	[$CHBr_3$] (10^{12} molecule cm^{-3})	[N_2O] (10^{13} molecule cm^{-3})	$k(T)$ (10^{-10} cm^3 $molecule^{-1}$ s^{-1})
Ar	32(3)	4.3(6)	6.2(9)	0.6 – 25.7	2.0(1)
Ar	32(3)	4.3(6)	6.3(9)	0.1 – 4.5	1.9(1)
Ar	32(3)	4.3(6)	3.0(5)	0.1 – 8.7	1.7(1)
Ar	32(3)	4.3(6)	3.0(5)	1.0 – 15.1	1.8(1)
Ar	32(3)	4.3(6)	3.0(5)	1.0 – 15.1	2.1(1)
Ar	32(3)	4.3(6)	3.0(5)	1.0 – 15.1	1.8(1)
Ar	32(3)	4.3(6)	6.0(9)	0.4 – 6.5	1.7(1)
Ar	32(3)	4.3(6)	6.0(9)	0.4 – 6.5	1.5(1)
Ar	32(3)	4.3(6)	6.0(9)	0.4 – 6.5	2.2(1)
				**Average**	**1.8(1)**
Ar	43(7)	7.0(1.7)	6.0(1.4)	1.4 – 14.9	2.5(2)
Ar	43(7)	7.0(1.7)	5.5(1.3)	1.0 – 14.9	2.5(1)
Ar	43(7)	7.0(1.7)	5.5(1.3)	1.0 – 15.1	2.6(1)
				**Average**	**2.5(2)**
Ar	47(5)	7.9(1.2)	3.0(5)	1.0 – 14.8	2.6(1)
Ar	47(5)	7.9(1.2)	3.0(5)	1.0 – 14.9	2.3(1)
Ar	47(5)	7.9(1.2)	3.0(5)	1.0 – 14.9	2.2(1)
				**Average**	**2.4(1)**
Ar	51(8)	10.3(2.5)	10.5(2.6)	1.0 – 15.0	2.3(1)
Ar	51(8)	10.3(2.5)	10.5(2.6)	1.0 – 15.0	1.9(1)
Ar	51(8)	10.3(2.5)	10.5(2.6)	1.0 – 15.0	1.3(1)
				**Average**	**1.9(1)**
Ar	54(5)	10.8(1.5)	4.0(6)	1.0 – 13.6	2.1(1)
Ar	54(5)	10.8(1.5)	4.0(6)	1.0 – 13.6	2.2(1)
Ar	54(5)	10.8(1.5)	4.0(6)	1.0 – 13.6	2.0(1)
				**Average**	**2.1(1)**

Ar	58(5)	11.5(1.4)	4.4(5)	1.1 – 14.9	2.0(1)
Ar	58(5)	11.5(1.4)	4.4(5)	1.1 – 14.9	2.1(1)
Ar	58(5)	11.5(1.4)	4.4(5)	1.1 – 14.9	2.1(1)
				**Average**	**2.1(1)**
Ar	73(3)	11.3(0.8)	17.0(1.2)	1.0 – 15.1	1.4(1)
Ar	73(3)	11.3(0.8)	17.0(1.2)	0.9 – 15.0	1.3(1)
Ar	73(3)	11.3(0.8)	17.0(1.2)	1.0 – 15.1	1.6(1)
				**Average**	**1.4(1)**
Ar	86(4)	5.1(0.4)	23.0(1.8)	1.4 – 17.4	0.9(1)
Ar	86(4)	5.1(0.4)	23.0(1.8)	1.3 – 15.1	1.1(1)
Ar	86(4)	5.1(0.4)	23.0(1.8)	1.3 – 15.2	1.2(1)
				**Average**	**1.1(1)**
Ar	110(4)	8.5(0.6)	33.0(2.5)	1.9 – 15.2	0.8(1)
Ar	110(4)	8.5(0.6)	35.0(2.6)	1.9 – 15.1	0.9(1)
Ar	110(4)	8.5(0.6)	35.0(2.6)	1.9 – 15.2	0.8(1)
				**Average**	**0.8(1)**

B. Capture Theory Calculations

For barrierless reactions where the rate coefficient of a reaction is governed by the long-range attractive forces, the gas kinetic limit can be approximated from classical capture theory (CCT). This capture theory model was initially developed to rationalise the behaviour of ion-molecule reactions and has been adapted for neutral-neutral reactions.[7–9] From simple collision theory (CT), the reaction rate coefficient is given by the product of the collision cross-section, σ, and the average velocity, v (E 1).

$$k(T) = \sigma v \qquad \text{E 1}$$

In the CT model, σ is calculated from the impact parameter (the sum of the radii of the two molecules). However, the CCT model differs in that σ is estimated to a good first-order approximation from an r^{-6} potential due to dipole–dipole (*D–D*), dipole–induced-dipole (*D–iD*), and London dispersion (Disp) intermolecular forces. This results in an expression for the reaction rate coefficient given by E 2 that depends on the Boltzmann constant, k_B, temperature, T, a gamma function ($\Gamma(2/3) = 1.533$), reduced mass of the collision partners, μ, and a sum of coefficients that describes the magnitude of the intermolecular forces between the molecules, C_6.

$$k(T) = \pi \left(\frac{2C_6}{k_B T}\right)^{\frac{1}{3}} \Gamma\left(\frac{2}{3}\right) \left(\frac{8k_B T}{\pi\mu}\right)^{\frac{1}{2}} \qquad \text{E 2}$$

The coefficient C_6 is given by E 3, which is a sum of the contributions of the dipole–dipole (*D–D*) coefficient, C_6^{D-D}, dipole–induced-dipole (*D–iD*) coefficient, C_6^{D-iD}, and London dispersion (Disp) coefficient C_6^{Disp}.

$$C_6 = C_6^{D-D} + C_6^{D-iD} + C_6^{Disp} \qquad \text{E 3}$$

These coefficients are described by equations E 4-E 6 for C_6^{D-D}, C_6^{D-iD}, and C_6^{Disp}, respectively.

$$C_6^{D-D} = \frac{2}{3}\frac{\mu_1^2\mu_2^2}{k_B T(4\pi\varepsilon_0)^2} \quad \text{E 4}$$

$$C_6^{D-iD} = \frac{\mu_1^2\alpha_2 + \mu_2^2\alpha_1}{4\pi\varepsilon_0} \quad \text{E 5}$$

$$C_6^{Disp} = \frac{3}{2}\alpha_1\alpha_2\frac{I_1 I_2}{I_1 + I_2} \quad \text{E 6}$$

Here, the coefficients depend on the dipole moment, μ_1 and μ_2, the polarizability, α_1 and α_2, and the ionisation potential, I_1 and I_2, of molecules 1 and 2. Therefore, the parameters provided in

Table **S3:** were used to calculate $k(T)$ for the CH + N_2O reaction for comparison with experimental and theoretical reaction rate coefficients (Figure 3 of the main text).

Table S3: Parameters used in the calculation of $k(T)_{CCT}$ for the reaction between CH and N_2O.

	**Dipole Moment**		**Polarizability**	**Ionisation Energy**	
**Molecule**	**(Debye)**	**(10^{-30} C m)**	**(10^{-30} m^3)**	**(eV)**	**(10^{-18} J)**
N_2O	0.161[10]	0.54	3.00[11]	12.89[12]	2.1
CH	1.46	4.87[13]	2.40[14]	10.64	1.70[15]

[10]Nelson *et al.*; [11]Onley *et al.*; [12]NIST; [13]Phelps & Dalby; [14]Manohar & Pal; [15]Herzberg & Johns

C. Electronic Structure Calculations

Table S4: Optimised cartesian coordinates at the M06-2X-D3/aug-cc-pV(Q+d)Z level of theory for all species along the reaction PES for the CH + N_2O reaction.

**Molecule**	**Atom**	**x**	**y**	**z**
CH	C	0.000000	0.000000	0.159336
	H	0.000000	0.000000	-0.956016
N_2O	N	0.000000	0.000000	-0.075443
	N	0.000000	0.000000	-1.185507
	O	0.000000	0.000000	1.103331
N_2	N	0.000000	0.000000	0.542712
	N	0.000000	0.000000	-0.542712
HCO	C	0.061645	0.579599	0.000000
	H	-0.863033	1.211424	0.000000
	O	0.061645	-0.586127	0.000000
NO	N	0.000000	0.000000	-0.605594
	O	0.000000	0.000000	0.529895
HCN	C	0.000000	0.000000	-0.494477
	N	0.000000	0.000000	0.646725
	H	0.000000	0.000000	-1.560214

HNC	N	0.000000	0.000000	0.426088
	C	0.000000	0.000000	-0.734312
	H	0.000000	0.000000	1.423256
P1INT1	C	-1.798244	-0.654630	-0.000003
	H	-2.538964	0.172647	0.000024
	N	1.609912	-0.460576	0.000002
	N	0.675702	0.126744	0.000000
	O	-0.333858	0.761494	-0.000002
P1INT2	C	-0.655824	0.413326	-0.112142
	H	-0.815804	1.447481	-0.439257
	N	1.617341	-0.296230	-0.211807
	N	0.745725	0.220685	0.349013
	O	-1.473839	-0.424828	0.018959
P1TS1	C	-1.346847	-0.665061	-0.151652
	H	-1.849289	-0.583107	0.828940
	N	1.530310	-0.308416	0.025290
	N	0.476189	0.045302	-0.001859
	O	-0.514390	0.801908	-0.010381
P1TS2	C	-0.776357	0.432208	-0.142571
	H	-1.049811	1.477206	-0.348416
	N	1.593952	-0.403056	-0.165127
	N	0.891178	0.378760	0.258733
	O	-1.460995	-0.487548	0.068575
P2INT1	N	0.257089	0.071695	0.000000
	N	-0.954352	-0.794147	-0.000001
	O	1.456987	-0.005142	-0.000002
	C	-0.888274	0.596739	0.000003
	H	-1.445412	1.517871	0.000006
P2Ts1	N	-0.289290	0.079629	0.118750
	N	1.023741	-0.750342	-0.026587
	O	-1.481813	-0.030927	-0.056185
	C	0.881771	0.573049	-0.013348
	H	1.422721	1.504115	-0.115577
P3TS1	N	1.605097	0.337120	-0.055239
	N	0.634456	-0.182341	0.102630
	O	-0.545962	-0.606610	-0.045182
	C	-1.696508	0.402413	-0.000319
	H	-1.130128	1.354950	0.031632
P4TS1	N	0.291705	0.096579	-0.424192
	N	-0.891071	-0.651475	-0.018474
	O	1.360350	-0.034954	0.208724
	C	-0.930564	0.688595	0.054525
	H	-1.103848	0.032334	1.101721

Table S5: Rotational constants (cm^{-1}) and unscaled vibrational frequencies (cm^{-1}) calculated at the M06-2X-D3/aug-cc-pV(Q+d)Z level of theory for all species in the reaction of CH + N_2O.

Molecule	Rotational Constants (cm^{-1})	Unscaled Vibrational Frequencies (cm^{-1})
CH	14.575	2883.18
N_2O	0.430	650.46, 650.46, 1364.96, 2423.20
N_2	2.044	2523.60
HCO	24.121, 1.520, 1.430	1105.81, 1998.12, 2721.65
NO	1.751	2071.6
HCN	1.511	788.08, 788.08, 2255.64, 3467.20
HNC	1.535	530.78, 530.78, 2149.53, 3820.09
P1INT1	0.956, 0.188, 0.157	79.60, 144.71, 152.36, 623.76, 631.51, 902.32, 1311.24, 2444.26, 2936.65
P1INT2	1.455, 0.192, 0.179	115.86, 248.14, 514.32, 733.52, 1003.97, 1344.14, 1876.66, 1954.84, 3058.20
P1TS1	0.926, 0.254, 0.204	-613.56, 277.11, 350.34, 533.67, 599.47, 1086.23, 1216.98, 2299.84, 2978.26
P1TS2	1.186, 0.185, 0.166	-527.04, 55.53, 285.87, 419.91, 818.65, 1231.06, 1936.93, 1981.34, 3004.92
P2INT1	1.098, 0.284, 0.226	378.22, 407.20, 541.83, 730.95, 969.93, 1215.51, 1280.63, 1963.57, 3279.68
P2TS1	1.175, 0.269, 0.220	-526.68, 411.25, 497.89, 826.41, 905.91, 1195.47, 1349.08, 1904.83, 3215.97
P3TS1	1.417, 0.204, 0.179	-1152.09, 211.82, 323.12, 556.80, 606.71, 913.38, 1311.28, 2197.41, 2931.89
P4TS1	1.040, 0.291, 0.258	-1626.41, 357.12, 447.83, 585.72, 886.08, 1053.73, 1348.93, 1424.50, 2311.07

Table S6: Electronic energies (CCSD(T)/aug-cc-pV(Q+d)Z energy ZPVE scaled and corrected, Hartree) for all species and the respective relative energies (kJ mol^{-1}) of all stationary points along the reaction PES.

Molecule	Electronic Energy (ZPVE Scaled and Corrected, Hartree)	Relative Energy (ZPVE Scaled and Corrected, kJ mol^{-1})
CH	-38.4130420	-
N_2O	-184.4542910	-
CH + N_2O	-	0.000000000
N_2	-109.4008773	-
HCO	-113.7074789	-
NO	-129.7529209	-
HCN	-93.2862971	-
HNC	-93.2635915	-
P1INT1	-222.8719486	-12.1182578
P1INT2	-223.0613622	-509.4236646
P1TS1	-222.8520826	40.0399252
P1TS2	-223.0607486	-507.8126578
P2INT1	-222.9168101	-129.9021261
P2TS1	-222.9189261	-135.4576841
P3TS1	-222.8518438	40.6668946
P4TS1	-222.8352949	84.1160315
N_2 + HCO	-	-632.8064116
NO + HCN	-	-451.2840675
NO + HNC	-	-391.6705147

References

(1) Rowe, B. R.; Canosa, A.; Heard, D. E. *Uniform Supersonic Flows in Chemical Physics*; WORLD SCIENTIFIC (EUROPE), 2021. https://doi.org/10.1142/q0324.

(2) Potapov, A.; Canosa, A.; Jiménez, E.; Rowe, B. Uniform Supersonic Chemical Reactors: 30 Years of Astrochemical History and Future Challenges. *Angewandte Chemie International Edition* **2017**, *56* (30), 8618–8640. https://doi.org/10.1002/anie.201611240.

(3) Driver, L.; Douglas, K.; Lucas, D. I.; Guillaume, T.; Lehman, J. H.; Kapur, N.; Heard, D. E.; de Boer, G. N. Developing a Predictive Model for Low-Temperature Laval Nozzles with Applications in Chemical Kinetics. *Physics of Fluids* **2024**, *36* (7), 076128. https://doi.org/10.1063/5.0216622.

(4) Lucas, D. I.; Guillaume, T.; Heard, D. E.; Lehman, J. H. Design and Implementation of a New Apparatus for Astrochemistry: Kinetic Measurements of the CH + OCS Reaction and Frequency Comb Spectroscopy in a Cold Uniform Supersonic Flow. *The Journal of Chemical Physics* **2024**, *161* (9), 094203. https://doi.org/10.1063/5.0220774.

(5) le Picard, S. D.; Canosa, A.; Rowe, B. R.; Brownsword, R. A.; Smith, I. W. M. Determination of the Limiting Low Pressure Rate Constants of the Reactions of CH with N_2 and CO: A CRESU Measurement at 53 K. *J. Chem. Soc., Faraday Trans.* **1998**, *94* (19), 2889–2893. https://doi.org/10.1039/A803930E.

(6) le Picard, S. D.; Canosa, A. Measurement of the Rate Constant for the Association Reaction CH + N_2 at 53 K and Its Relevance to Triton's Atmosphere. *Geophysical Research Letters* **1998**, *25*, 485–488. https://doi.org/10.1029/98GL50118.
(7) Clary, D. C. Fast Chemical Reactions: Theory Challenges Experiment. *Annual Review of Physical Chemistry* **1990**, *41* (Volume 41,), 61–90. https://doi.org/10.1146/annurev.pc.41.100190.000425.
(8) Clary, D. C.; Haider, N.; Husain, D.; Kabir, M. Interstellar Carbon Chemistry: Reaction Rates of Neutral Atomic Carbon with Organic Molecules. *The Astrophysical Journal* **1994**, *422*, 416. https://doi.org/10.1086/173737.
(9) Tsikritea, A.; Diprose, J. A.; Softley, T. P.; Heazlewood, B. R. Capture Theory Models: An Overview of Their Development, Experimental Verification, and Applications to Ion–Molecule Reactions. *The Journal of Chemical Physics* **2022**, *157* (6), 060901. https://doi.org/10.1063/5.0098552.
(10) Nelson, R. D.; Lide, D. R.; Maryott, A. A. Selected Values of Electric Dipole Moments for Molecules in the Gas Phase: **1967**. https://doi.org/10.6028/NBS.NSRDS.10.
(11) Olney, T. N.; Cann, N. M.; Cooper, G.; Brion, C. E. Absolute Scale Determination for Photoabsorption Spectra and the Calculation of Molecular Properties Using Dipole Sum-Rules. *Chemical Physics* **1997**, *223* (1), 59–98. https://doi.org/10.1016/S0301-0104(97)00145-6.
(12) Informatics, N. O. of D. and. *NIST Chemistry WebBook*. https://webbook.nist.gov/chemistry/ (accessed 2025-08-20).
(13) Phelps, D. H.; Dalby, F. W. Experimental Determination of the Electric Dipole Moment of the Ground Electronic State of CH. *Phys. Rev. Lett.* **1966**, *16* (1), 3–4. https://doi.org/10.1103/PhysRevLett.16.3.
(14) Manohar, P. U.; Pal, S. Dipole Moments and Polarizabilities of Some Small Radicals Using Constrained Variational Response to Fock-Space Multi-Reference Coupled-Cluster Theory. *Chemical Physics Letters* **2007**, *438* (4), 321–325. https://doi.org/10.1016/j.cplett.2007.02.075.
(15) Herzberg, G.; Johns, J. W. C. New Spectra of the CH Molecule. *The Astrophysical Journal* **1969**, *158*, 399–418. https://doi.org/10.1086/150202.
